# "Sleepiness" is serious in adolescence: Two surveys of 3235 Canadian students

**DOI:** 10.1186/1471-2458-6-116

**Published:** 2006-05-02

**Authors:** Edward S Gibson, AC Peter Powles, Lehana Thabane, Susan O'Brien, Danielle Sirriani Molnar, Nik Trajanovic, Robert Ogilvie, Colin Shapiro, Mi Yan, Lisa Chilcott-Tanser

**Affiliations:** 1 Department of Clinical Epidemiology and Biostatistics, Faculty of Health Sciences, McMaster University, 1200 Main Street West, Hamilton, Ontario, L8N 3Z5, Canada; 2 Faculty of Medicine, McMaster University, St. Joseph's Healthcare, 50 Charlton Street, Hamilton, Ontario, L8N 4A6, Canada; 3 Centre for Evaluation of Medicines, St. Joseph's Healthcare, 105 Main Street East, Level P1, Hamilton Ontario, L8N 1G6, Canada; 4 Ancaster High School, Hamilton Wentworth District School Board, 374 Jerseyville Road West, Hamilton, Ontario, L9G 3K8, Canada; 5 Department of Psychology, Brock University, 500 Glenridge Avenue, St. Catharines, Ontario, L2S 3A1, Canada; 6 Sleep and Alertness Clinic, Toronto Western Hospital, 399 Bathurst Street, Toronto, Ontario, M5T 2S8, Canada; 7 Department of Psychiatry, Faculty of Medicine, University of Toronto, Toronto Western Division, 399 Bathurst Street, Toronto, Ontario, M5T 2S8, Canada; 8 Department of Statistics and Actuarial Sciences, University of Waterloo, Waterloo, Ontario, N2L 3G1, Canada; 9 Central-West Sleep Laboratories, 139 Grand River Street North, Paris, Ontario, N3L 2M4, Canada

## Abstract

**Background:**

Evidence is growing that sleep problems in adolescents are significant impediments to learning and negatively affect behaviour, attainment of social competence and quality of life. The objectives of the study were to determine the level of sleepiness among students in high school, to identify factors to explain it, and to determine the association between sleepiness and performance in both academic and extracurricular activities

**Methods:**

A cross-sectional survey of 2201 high school students in the Hamilton Wentworth District School Board and the Near North District School Board in Ontario was conducted in 1998/9. A similar survey was done three years later involving 1034 students in the Grand Erie District School Board in the same Province. The Epworth Sleepiness Scale (ESS) was used to measure sleepiness and we also assessed the reliability of this tool for this population. Descriptive analysis of the cohort and information on various measures of performance and demographic data were included. Regression analysis, using the generalised estimating equation (GEE), was utilized to investigate factors associated with risk of sleepiness (ESS>10).

**Results:**

Seventy per cent of the students had less than 8.5 hours weeknight sleep. Bedtime habits such as a consistent bedtime routine, staying up late or drinking caffeinated beverages before bed were statistically significantly associated with ESS, as were weeknight sleep quantity and gender. As ESS increased there was an increase in the proportion of students who felt their grades had dropped because of sleepiness, were late for school, were often extremely sleepy at school, and were involved in fewer extracurricular activities. These performance measures were statistically significantly associated with ESS. Twenty-three percent of the students felt their grades had dropped because of sleepiness. Most students (58–68%) reported that they were "really sleepy" between 8 and 10 A.M.

**Conclusion:**

Sleep deprivation and excessive daytime sleepiness were common in two samples of Ontario high school students and were associated with a decrease in academic achievement and extracurricular activity. There is a need to increase awareness of this problem in the education and health communities and to translate knowledge already available to strategies to address it.

## Background

Evidence is growing that sleep problems in adolescents are significant impediments to learning, cognition and memory. Sleep problems negatively affect behaviour, attainment of social competence and quality of life and are more prevalent than realized by education and health professionals. There is little indication of any degree of awareness in the education or health care systems in Canada sufficient to initiate appropriate interventions.

Despite extensive research over the past decades, sleep regulatory mechanisms are still not fully understood. Adolescents are faced with developmental changes affecting these mechanisms as well as psychosocial changes. Carskadon has shown that adolescents require at least 8.5 hours sleep [1]. Carskadon and Wolfson [2] followed 40 students passing through grade 9 to 10 to study the effect of earlier school start time (from 0825 hours to 0720) on sleep patterns. Early start was associated with significant sleep deprivation and daytime sleepiness; students went to bed later and slept less. Significant physiological findings identified in the students in grade 10 were later onset of melatonin secretion, shortened sleep latency and marked increase in incidence of REM (rapid eye movement) sleep. Carskadon's [3] research on the sleep-wake homeostatic process and its interaction with the circadian biological timing system shows evidence of changes during pubertal development which alter sleep patterns substantially. These findings suggest the bioregulatory mechanisms controlling sleep in adolescents delay sleep onset (sleep phase delay) and hence normal sleep times are truncated by societal requirements such as early school schedules.

Assessing academic achievement, Randazzo [4] found children randomized to one night of five hours sleep showed impaired higher cognitive function such as verbal creativity and abstract thinking, although their routine performance was maintained, compared to those randomized to eleven hours sleep. Carskadon and Wolfson [5-7] showed the relationship between adolescent sleep and performance. Wolfson [8], surveyed 3,120 high school students, finding those who reported grades as C, D or F had 25 minutes less weeknight sleep than those who reported A or B grades. However, one study by Eliasson [9] failed to demonstrate a clear association between sleep quantity and academic achievement.

With respect to behaviour Dahl [10] notes, "the empiric data to directly address the effects of sleep loss or disruption on children's cognitive function are quite sparse. However, a wide range of clinical and observational data support a general picture that inadequate sleep results in tiredness, difficulties with focused attention, low threshold to express negative affect (irritability and easy frustration) and difficulty modulating impulses and emotions. These symptoms may resemble Attention Deficit Hyperactivity Disorder." Morrison [11] found adolescents reporting sleep problems showed more anxious, depressed, inattentive, and conduct disorder behaviour than those who had no, or occasional, sleep problems. In a study of 49 children, Aronen [12] found that low sleep time was significantly associated with teacher reports of aggressive and delinquent behaviour and social and attention problems.

In order to justify further research and initiatives to address the problem in teenage students it is necessary to ask the questions, how prevalent is "sleepiness" and does it have a significant impact on curricular or extracurricular activities? The hypothesis was that adolescents, for a variety of reasons, are not getting adequate sleep. If this were the case, then their performance, both curricular and extracurricular, would be affected. The surveys in this study were designed to answer these questions and examine possible associated factors in representative samples of Ontario high school students.

## Methods

Due to the lack of baseline data about sleep in Canadian adolescent students, focus groups of students, teachers, parents and school administrators were convened to identify problem areas. "Sleepiness" was identified as a significant problem and the major obstacle to increasing awareness was identified as the lack of information about the extent and impact of sleepiness. The focus groups, along with sleep researchers and clinicians, identified questions that they felt needed an answer and were specific to this population. No questionnaires were identified that fulfilled the requirements or that could be completed within a proscribed 15–20 minutes.

Permission was granted by the Hamilton Wentworth District School Board (H/W) to conduct an anonymous voluntary survey to elicit this information in two secondary schools in the autumn of 1998. In the spring of 1999 a rural secondary school from the Near North Board was also included. This school was included with H/W for analysis. The schools were located in a multicultural inner city neighbourhood, a middle class neighbourhood and a rural community. The student population in the three schools was 3010.

The self completed survey contained items dealing with students' school and extracurricular functioning, bed and wake times (sleep hours were calculated as the difference between a student's report of "usual" bed and wake times on weeknights), bedtime routines, daytime periods of feeling "really sleepy", questions eliciting symptoms suggestive of sleep disorders and demographic information. The Epworth Sleepiness Scale [13] was included, a scale that has been used extensively as a measure of daytime sleepiness. This scale had functioned well in the focus groups and in test-retest reliability. Scores for excessive daytime sleepiness range from none (0) to extreme (24). The study questionnaire was reviewed for face validity by the partners in education and sleep clinicians. Test-retest reliability was determined using 30 students to complete the questionnaire on two occasions two weeks apart.

Staff, students and parents of the schools involved were informed of the nature and purpose of the study. The homeroom teacher administered questionnaires at the same hour to each class available at that time.

As a result of this initial survey, and in particular the finding that a significant number of students indicated an unrecognized sleep disorder, a second survey, using the same questionnaire, was conducted in 2001 in Grand Erie District School Board (G/E). The four schools were in a small city, a rural town and a larger city with a vocational school and another with a large number of First Nation's students. The student population in the four schools was 3650.

Start times for all schools varied from 8:15 to 8:45 A.M.

It was felt that concordance of results, from two large populations completing identical questionnaires three years apart, would serve to increase confidence in the results.

### Sample size

A primary objective of the study was to determine the factors that explain sleepiness as measured by the Epworth Sleepiness Scale [13] using regression techniques. The total sample size in the study was 3235 students. Simulation studies demonstrate that modeling binary outcomes requires 10 to 15 events per predictor to produce stable estimates [14,15]. Note that our multivariable analyses included at mostsix predictors. Therefore, the large sample of 3235 that we used was sufficient to address both primary and secondary objectives of the study.

### Analysis

The results of participants' demographic, sleepiness and performance outcomes were summarized using descriptive summary measures: expressed as mean (standard deviation [SD]) for continuous variables with symmetric distribution or median (minimum [min] - maximum [max]) for those with non-symmetric distribution, and number (percent) for categorical variables. All statistical tests were performed using two-sided tests at the 0.05 level of significance. We used generalized estimating equations (GEE) [16,17] assuming an exchangeable structure for the correlation matrix, to model both binary and continuous outcomes. Unlike ordinary regression analysis technique, GEE allows accounting for possible correlation of responses from students within the same school. The model covariates included gender, age, sleep hours, bedtime habits (consistent bedtime routine, up late to study, drinking caffeinated beverages after 6 P.M., smoking before bed, eating before bed). The GEE results were expressed as coefficient, corresponding standard error, odds ratio and corresponding 95% confidence interval (CI) and associated p-value for test of significance. P-values were reported to three decimal places with p-values less than 0.001 reported as p < 0.001. We also reported the estimate of the intra-class correlation coefficient (ICC) for multivarible models to determine the within-school clustering effect. We assessed multicolinearity using the variance inflation factor (VIF), which measures the extent to which the variance of the model coefficients will be inflated (because of the correlation of the variable with other predictor variables) if that variable is included in the model. Variables with VIF > 10 were considered colinear and were excluded from the analysis [18]. We examined the residuals to assess model assumptions. Goodness-of-fit for GEE models was performed using Hosmer-Lemeshov test (H-LT) [19]. All analyses were performed using SAS version 9.2 (Cary, North Carolina).

### Ethical considerations

This study was conducted according to the Ethical Conduct for Research Involving Human Tri-Council Policy Statement [20]. We applied several security safeguards in the data access, handling, and storage. There were no personal identifiers recorded in the database. Hamilton Wentworth District School Board (H/W) granted us permission to conduct an anonymous voluntary survey to elicit this information in two secondary schools in the autumn of 1998 and a rural secondary school from the Near North Board in the spring of 1999. Grand Erie District School Board (G/E) also granted similar permission to conduct the survey in three schools in 2001. The study received research ethics approval from the Research Ethics Board of Brock University. All participants or parents and guardians provided informed consent prior to participation.

## Results

### Participation rates

Complete questionnaires were submitted by 2201(73%) of the students in the schools from the Hamilton Wentworth District School Board and the Near North Board (1177 male, 1024 female). In the Grand Erie Board questionnaires were completed by 1034(28%) of the students (467 male, 567 female). The distribution across age and grade was similar for both Boards, and the distribution by bedtime habits and performance is shown in Table 1.

### Test-retest reliability

Test retest reliability for the questions used in the survey showed ICC values from 0.66 to 0.89. The ICC for the Epworth scale was 0.88.

### Prevalence of sleepiness

Mean Epworth scores from the individual schools did not show any statistically significant difference and Epworth scores were identical for both Boards, with a typical normal distribution and mean (SD) of 8.75 (4.35) for H/W and 8.73 (4.33) for G/E. Excessive daytime sleepiness (ESS > 10) was reported by 42% of the Grand Erie students and 41% of the Hamilton Wentworth students.

**Figure 1:**
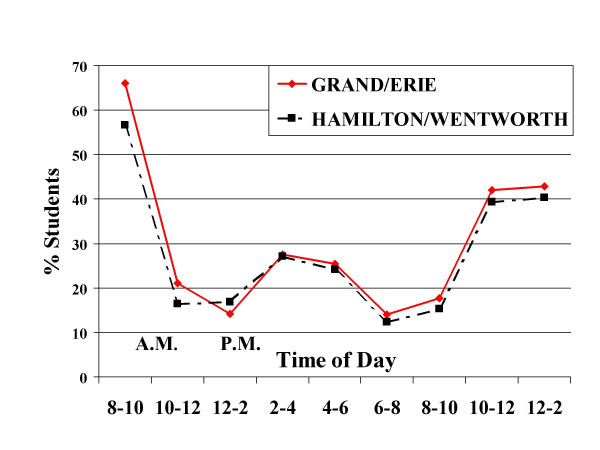
Per cent students feeling "really sleepy" at different times of day.

Morning sleepiness was common, with the largest percentage of students, 58–68%, feeling "really sleepy" between 8 and 10 A.M. (Figure 1). Otherwise the distribution showed a typical circadian pattern throughout the day, with identical responses from both Boards. Seventy-three per cent of these students had performance measures 3–5% less satisfactory than their peers, however, the other 27% were also very sleepy between 10 and 12 in the morning and showed a 46–220% change in proportion of those who had performance problems (grades down – increase 81%; lates – increase 46%; extremely sleepy at school – increase 220%; less activities – increase 98%; miss social/sports – increase 73%). On a 7-point Likert scale, where 1 represented being very tired and groggy on awaking, 51% and 57% from the two boards scored 3 or less, and when asked whether it was difficult to wake in the morning, 41% and 47% of the students responded "often".

### Factors associated with sleepiness

Eight factors were tested in the model, age, gender, weeknight sleep hours, consistent bedtime pattern, staying up late to study, drinking caffeinated beverages after 6 P.M., smoking before bed and eating before bed. As shown in Tables 2 and 3, five factors showed a statistically significant relationship with the Epworth score. For students with ESS >10 mean Epworth scores associated with gender for H/W and G/E respectively were male 13.89 and 13.84, and female 13.56 and 13.49.

Approximately 70% of the students in both Boards had less than 8.5 hours weeknight sleep with an identical distribution by hours of sleep. Overall mean (SD) sleep hours were 7.77 (1.16) for H/W and 7.80 (1.09) for G/E. Comparing means for ESS >10 and ESS =<10, mean sleep hours were 7.58 (1.19) and 7.86 (1.13) for H/W and 7.57 (1.18) and 7.91 (1.03) for G/E respectively.

### Age effects

Mean (SD) age for males in H/W was 16.2 (1.96) years and for G/E 16.3 (1.46), for females it was 16.3 (2.26) for H/W and 16.1 (1.37) for G/E. Average hours of sleep for all students decreased by about 45 minutes from age 14 to 18 years, with bedtimes averaging 60 minutes later and wake times 15 minutes later. On weekends, bed times and wake times were later with sleep times averaging about 1 hour and 20 minutes more than on weeknights. As age increased there were decreases in the per cent of students who went to sleep before 12:00 P.M. (95.7% at age 14, 62.2% at age 18) and increases for those who awoke after 7:30 A.M. (13.7% a age 14, 36.2% at age 18).

### Sleep and performance

Our analysis showed that the five performance measures, late for school past month, often extremely sleepy at school, grades dropped because of sleepiness, decreased daytime activities, miss social, sport event or work in past month because of sleepiness, were significantly associated with the risk of sleepiness (Table 4.) As Epworth scores increased from 0–24 there was a linear increase in per cent of students who reported a problem with a performance measure. "Lates" showed a similar linear relationship, but even with no excessive daytime sleepiness (ESS = 0), 20% of the students reported being late in the previous month. Positive response to the question "Are you often extremely sleepy at school?" was 13.4% for H/W and 14.45 for G/E. Table 5 shows the distribution of response to the question "Are you often extremely sleepy at school?" by performance indicators for both H/W and G/E. Overall the majority who reported being "Yes" also reported their grades going down and being late for school. While this observation does not necessarily imply cause-effect relationship, it does indicate the possibility of a strong association between "sleepiness" and performance.

## Discussion

The majority of students (70%) were mildly to severely sleep deprived based on Carskadon's [1] finding that adolescents require at least 8.5 hours of weeknight sleep. Excessive daytime sleepiness was common, with 40.9% of H/W students and 41.9% of G/E students reporting ESS score greater than 10. Five factors, gender, hours of weeknight sleep, consistent bedtime pattern, up late to study and drinking caffeineated beverages after 6 P.M. were statistically significantly associated with ESS >10.

The impact on performance was important, with a statistically significant association between ESS > 10 and five performance measures, grades dropping, lates, being extremely sleepy at school, having decreased extracurricular activities and missing social or sport events or work. Further, there was a linear relationship between ESS scores and performance measures.

Morning sleepiness was common as the largest proportion of students reported being very sleepy between 8 and 10 A.M. (Figure 1). In addition, a significant proportion of students, even those with low Epworth scores, reported they were late for school in the previous month. Many students reported difficulty waking in the morning and being tired on waking These findings likely reflect the effect of "sleep phase delay" and corresponding change in circadian rhythms which begins in adolescence [2,3], confirming that adolescents are often not ready to function early in the morning. The students also went to sleep later on weekends, thus reinforcing the sleep phase delay. With sleep phase delay, adolescents are really in a "sleep" state early in the morning, but our data show that many of these students manage to perform reasonably well. For those who were very sleepy only between 8 and 10 A.M. performance measures were just 3–5% less satisfactory than their peers, but those who were also very sleepy between 10 A.M. and 12 P.M. exhibited increases in proportion of performance problems of 46–220%. Sleep phase delay is a physiological phenomenon with onset at puberty [3]. Parents and educators need to know that adolescents' tendency to go to bed late and wake late is normal, and this must be considered in addressing sleep habits and in academic scheduling and transportation.

Early morning is a difficult time for many students to absorb and remember complicated material. This conforms to the findings of Hansen [21]; in a study of 60 high school seniors, students in early morning classes reported being wearier, being less alert and having to expend greater effort. All students performed better in the afternoon.

The 24.4% of Grand Erie and 22.7% of Hamilton Wentworth students who felt their grades had dropped because of sleepiness reported 26 and 27 minutes less weeknight sleep respectively than their peers. These times are comparable to the 25 minutes found in Wolfson's study [8]. The association of these modest sleep losses with lowered academic performance has now been demonstrated in three student samples. The potential significance of an alteration in performance associated with about 25 minutes extra sleep warrants further exploration.

Memory is critical to learning and adequate sleep is critical for memory. Memory is consolidated, and may be enhanced, during sleep after a learning exposure. The relationships between memory, learning and sleep have been outlined by Smith [22] who noted that declarative material (rote memorizing) is usually explicitly (consciously) learned, while procedural material (learning a novel cognitive or motor task) is usually implicitly (unconsciously) acquired. While declarative material does not seem very sensitive to sleep loss, procedural is very sensitive to sleep loss.

It is worrisome that educators continue to fail to recognize students who may have disrupted sleep patterns and that physicians fail to ask about sleep. Owen [23] found 44% of paediatricians did not routinely screen for sleep disorders in adolescents and underdiagnosis of sleep problems has been noted by a number of authors [24-29].

There are potential concerns with the study. Although the consistency of results from seven schools in different geographic and socioeconomic environments with a time interval of three years lends confidence to the findings, the data were based entirely on anonymous self-completed questionnaires and thus subject to responder bias. The potential problem of clustering (sampling seven schools) was addressed in the analysis. The ESS performed well in this survey; however the addition of a more recent validated sleep questionnaire for comparison would be desirable. Sleep times can be accurately measured using actigraphy; although this would be impractical for such a large sample, the use of a nested random sample of students would be valuable. There is evidence that sleep quality is a better measure for adequate sleep than sleep times; this needs to be explored. Student grades are regarded as highly confidential by the education system; the number of releases necessary to obtain these could be impractical. Again, the use of a random sample for comparison could be utilized. School "lates" records are accurately maintained in these jurisdictions and would be accessible. Measures of bedtime sleep habits would be enhanced by the use of parent questionnaires that would have a secondary benefit of focusing the attention of parents on appropriate ways to improve "sleep hygiene". A major deficit was the lack of a well validated questionnaire to identify sleep disorders in this population. It was the intent in the G/E survey to identify these students, but an unfortunate and unforeseen circumstance made that impossible. In addition, there was no clear differentiation between the environmental, physiological and behavioural causes of sleepiness as opposed to medical causes such as depression or Attention Deficit Disorders. This is an area of research that needs to be explored more thoroughly. Participation rates were different for the two Boards, 73% and 28%. Despite this the results were remarkably similar. The 28% rate in G/E reflects the difficulties of obtaining signed consent in the school system; however strategies were not in place for follow-up or reminders. This will need to be addressed in subsequent research.

The students were drawn from one of the most populous areas in Canada, so caution is needed in generalizing the results or applying them to other populations.

## Conclusion

"Sleepiness" and sleep deprivation in Ontario high school students are common and are associated with a significant detrimental effect on curricular and extracurricular activities. It is a problem that has been afforded little recognition and little effort to design interventions. Sleepiness is a symptom and is associated with multiple factors, among which are sleep deprivation, poor sleep hygiene, sleep phase delay, sleep disorders, societal demands, depression and Attention Deficit Disorders.

Sleep phase delay is an important and generally unrecognized factor in sleepiness and needs to be recognized as a normal and frequent occurrence in adolescence. There is a need for educators to be more aware of the impact of school start times and academic scheduling, and to consider sleep problems as potential factors in students who fail to achieve or who exhibit behavioural problems. While it may be administratively convenient to begin high school classes early, there is strong evidence in our data, supported by the literature, suggesting that later start times would be more appropriate for teens. This may also be a problem for post-secondary education. In a survey of 59 Canadian Universities [30] only 9(15%) started classes at 9:00 A.M., 37(63%) at 8:30 and 13(22%) at 8:00 A.M.

The data suggest potential indicators for students at risk may be falling asleep in class (extremely sleepy at school), falling grades, frequent lates and, from the literature, behavioural changes. Potential approaches to management are improvement in sleep hygiene, including recognition and strategies directed toward sleep phase delay as a normal accompaniment of adolescence, alteration of school scheduling, and identification and management of sleep disorders. There is a need for clinicians to be more alert to the possibility of sleep problems in their adolescent patients.

Perhaps the most significant impact of sleep deprivation may be on the secondary development of the brain that commences in puberty. The lag between attainment of sexual maturity and emotional development of high intensity feelings, such as risk taking, and the development of a set of neurobehavioural systems for self-control and affect regulation may be accentuated by sleep deprivation. For example the risk for addiction is high in this population. Relatively recent transdisciplinary research has begun to address these questions [31].

There is a substantial body of evidence concerning sleep problems in adolescence as clearly demonstrated by Millman's [32] extensive review of the literature. A major priority is the translation of this knowledge into strategies to resolve the problems [33]. It is critical that further research in this area directly and effectively involves the partners in education, students, parents, teachers, administrators, school trustees and governments as well as researchers and clinicians. Wahlstrom [34] has clearly defined the obstacles and approaches to initiating change in the education system. We need to pay attention.

## Competing interests

The author(s) declare that they have no competing interests.

## Authors' contributions

ESG was responsible for concept of the study, design of the study, obtaining funding, supervising the study and helped draft the manuscript. ACPP was involved in design of the study, consulting on sleep medicine, participating in focus groups, participating in drafting the paper. LT was principally responsible for statistical design and analysis and in drafting the paper. SO'B coordinated the schools participation, participated in the design of the study, focus groups and assisted in drafting the paper. DSS was responsible for design and coordination of the second phase of the study and participated in the statistical analysis and drafting of the paper. NT was involved in the focus groups, design of the study and participated in drafting the paper. RO was consultant to the second phase of the study, drafted the ethical requirements and obtained the approval of the ethics committee. He also was involved in drafting the paper. CS was involved in the concept of the study, consulted in sleep medicine and participated in reviewing the paper. MY was involved in the statistical design and analysis and drafting the statistical presentations in the paper. LCT was involved in the questionnaire development and was responsible for the focus groups as well as reviewing the paper.

All authors read and approved the final manuscript.

## Pre-publication history

The pre-publication history for this paper can be accessed here:



## Figures and Tables

**Table 1:** Distribution of students by bedtime habit frequency response of "often" and by performance.

		**Hamilton Wentworth**		**Grand Erie**	
		**All n(%)**	**ESS =>10**	**All n(%)**	**ESS =>10**

**n**		**2201**	**900**	**1034**	**433**

**Gender**	**M**	1177 (53.5)	462 (51.3)	467 (45.2%)	189 (43.6)
	**F**	1024 (46.5)	438 (48.7)	567 (54.8%)	244 (56.4)
**Bedtime habit**					
**Consistent pattern**		627 (28.5)	208 (23.1)	299 (28.9)	87 (20.1)
**Up late to study**		287 (13.0)	145 (16.1)	132 (12.8)	73 (16.9)
**Caffeine after 6 P.M.**		774 (35.2)	331 (36.7)	432 (41.8)	200 (46.2)
**Smoke before bed**		301 (13.7)	152 (16.9)	115 (11.1)	70 (16.2)
**Eat before bed**		577 (26.2)	264 (29.3)	285 (27.6)	154 (35.7)
**Performance**					
**Grades down**		501 (22.7)	302 (33.5)	252 (24.4)	154 (36.7)
**Late past month**		1037 (47.1)	504 (55.9)	404 (39.1)	228 (52.7)
**Extremely sleep at school**		245 (13.4)	205 (22.8)	149 (14.4)	108 (24.9)
**Decreased activities**		373 (16.9)	235 (26.1)	188 (18.2)	126 (29.1)
**Miss social sports**		434 (19.9)	270 (30.0)	218 (21.1)	138 (31.4)

**Table 2:** Univariable GEE results, region, age, gender, Epworth scores and bedtime habits.

**ESS Score Parameter**	**Odds Ratio (OR)**	**95% Confidence Interval**		**p value**
**Region**	0.9625	0.6192	1.4962	0.8652
Age (years)*	1.0820	0.9435	1.2409	0.2594
**Gender**	1.3844	1.1560	1.6578	0.0004
Weeknight sleep hours**	0.5911	0.4951	0.7057	<0.0001
**Consistent bedtime routine**	0.3965	0.3332	0.4719	<0.0001
**Up late to study**	1.6393	1.2921	2.0797	<0.0001
**Caffeine after 6 P.M.**	1.3263	1.2537	1.4031	<0.0001
**Smoke before bed**	1.2295	0.9677	1.5621	0.0909
**Eat before bed**	1.0847	0.9976	1.1794	0.0569

* OR calculated based on one year of age

** OR calculated based on one hour of weeknight sleep

**Table 3:** Multivariable GEE results – Epworth scores and bedtime habits.

**Variable**	**Odds Ratio**	**95%Confidence Interval**		**p value**
**Gender**	1.1042	1.0369	1.1759	0.0020
Weeknight sleep hours*	0.7328	0.6411	0.8376	<0.0001
**Consistent bedtime routine**	0.4554	0.3756	0.5523	<0.0001
**Up late to study**	1.4594	1.1667	1.8256	0.0009
**Caffeine after 6 P.M.**	1.2318	1.2064	1.2577	<0.0001
**Smoke before bed**	1.1841	0.9983	1.4045	0.0523
**Eat before bed**	1.0007	0.9632	1.0397	0.9727

* OR calculated based on one hour of weeknight sleep (ICC = 0.0018)

**Table 4:** Univariable GEE analysis: association between Epworth scores and performance measures.

**Variable**	**Odds Ratio**	**95% Confidence Interval**		**p value**
**Late**	1.1067	1.0878	1.1258	<0.0001
**Extremely sleepy**	1.2211	1.1940	1.2487	<0.0001
**Grades down**	1.1698	1.1436	1.1965	<0.0001
**Less daytime activities**	1.1610	1.1461	1.1761	<0.0001
**Miss social/sports**	1.1578	1.1373	1.1787	<0.0001

**Table 5:** Association of per cent of students reporting each performance measure as "yes" with frequency of being extremely sleepy at school

		**Total**	**Grades down n (%)**	**Lates n (%)**	**Activity down n (%)**	**Miss social/sports n (%)**
**All students**	**HW**	2201	497(22.6)	1037(47.1)	374(17.0)	440(20.0)
	**GE**	1034	252(24.3)	404(39.1)	189(18.3)	218(21.1)
**Extremely sleepy**						
**Often**	**HW**	295	157(53.2)	191(64.4)	126(43.1)	118(40.0)
	**GE**	149	86(57.7)	86(57.4)	76(50.7)	63(42.3)
**Sometimes**	**HW**	869	230(26.5)	453(52.1)	169(19.5)	202(23.2)
	**GE**	442	125(28.2)	203(45.9)	86(19.5)	110(24.9)
**Seldom**	**HW**	768	97(12.6)	304(39.6)	66(8.6)	96(12.5)
	**GE**	357	41(11.5)	95(26.6)	27(7.6)	44(12.4)
**Never**	**HW**	269	13(4.9)	89(33.2)	11(4.1)	15(5.6)
	**GE**	86	0(0.0)	21(24.4)	0(0.0)	1(1.2)
